# Streamlining the Analysis of Dynamic ^13^C-Labeling Patterns for the Metabolic Engineering of *Corynebacterium glutamicum* as l-Histidine Production Host

**DOI:** 10.3390/metabo10110458

**Published:** 2020-11-12

**Authors:** André Feith, Andreas Schwentner, Attila Teleki, Lorenzo Favilli, Bastian Blombach, Ralf Takors

**Affiliations:** 1 Institute of Biochemical Engineering, University of Stuttgart, Allmandring 31, 70569 Stuttgart, Germany; andre.feith@ibvt.uni-stuttgart.de (A.F.); andreas.schwentner@uni-ulm.de (A.S.); attila.teleki@ibvt.uni-stuttgart.de (A.T.); lorenzo.favilli@uni.lu (L.F.); 2 Institute of Microbiology and Biotechnology, Ulm University, Albert-Einstein-Allee 11, 89081 Ulm, Germany; 3 Microbial Biotechnology, Campus Straubing for Biotechnology and Sustainability, Technical University of Munich, Schulgasse 22, 94315 Straubing, Germany; bastian.blombach@tum.de

**Keywords:** design-build-test-learn (DBTL) cycle, metabolomics, strain engineering, l-histidine, *Corynebacterium glutamicum*, hydrophilic interaction chromatography (HILIC), quadrupole time-of-flight high-resolution mass spectrometer (QTOF-HRMS), pool influx kinetics, metabolic engineering

## Abstract

Today’s possibilities of genome editing easily create plentitudes of strain mutants that need to be experimentally qualified for configuring the next steps of strain engineering. The application of design-build-test-learn cycles requires the identification of distinct metabolic engineering targets as design inputs for subsequent optimization rounds. Here, we present the pool influx kinetics (PIK) approach that identifies promising metabolic engineering targets by pairwise comparison of up- and downstream ^13^C labeling dynamics with respect to a metabolite of interest. Showcasing the complex l-histidine production with engineered *Corynebacterium glutamicum* l-histidine-on-glucose yields could be improved to 8.6 ± 0.1 mol% by PIK analysis, starting from a base strain. Amplification of *purA*, *purB*, *purH,* and formyl recycling was identified as key targets only analyzing the signal transduction kinetics mirrored in the PIK values.

## 1. Introduction

Since the advent of synthetic biology, a steadily increasing number of tools for successful strain engineering have been developed. Recent advances in genome editing [1], creation of standardized bricks [2], adaptive laboratory evolution [3] targeted gene deletion CRISPR/Cas [4], gene silencing CRISPRi [5], and high-throughput screening [6] provide the ground for designing and testing multitudes of novel strains. Combining these tools with the intense application of robotics allows for the creation of a bunch of experimental data ready to qualify the constructs and select the best candidates for the next round of strain engineering.

The well-known design-build-test-learn (DBTL) cycle requires a knowledge-driven design strategy to start subsequent rounds of strain engineering. Enabling tools such as metabolite analysis [7] and bioinformatics [8] already exist. However, the question arises whether complementary technologies that allow for the identification of promising metabolic engineering targets via robust, easy-to-implement technologies only requiring a minimum of particular skills for application are still missing. Notably, such technologies should be suited to analyze the grand parameter space that today’s tools of strain engineering are modulating. Here, we investigated the applicability of ^13^C-labeled metabolomics for this purpose.

The standard approach for testing is to detect a target molecule, in most cases, the actual product, through techniques like gas or liquid chromatography (GC, LC) with UV absorbance or mass spectrometry (MS). A different approach, especially suited for high-throughput applications, is the use of biosensors via protein or transcript-based sensing of a target molecule [9]. However, the sole analysis of a target molecule can only tell the strain engineer if the corresponding product yield increased or decreased. With this strategy, failures cannot be explained, and possible metabolic bottlenecks cannot be identified. 

To extract more information out of engineered strains, the analysis of intracellular and extracellular metabolites, called metabolomics, has proven to be promising since the metabolome most closely reflects the actual cellular physiological state compared to the genome or proteome as underlying regulatory mechanisms [10,11,12]. Advanced GC- or LC-MS analytical methods need to be applied to either screen the whole-cell metabolite extracts in an untargeted fashion to acquire qualitative or semiquantitative information [13,14,15] or to quantify a targeted predefined subset of metabolites [11,13,15]. Typical systems used for this are hydrophilic interaction chromatography (HILIC) coupled to triple quadrupole tandem mass spectrometry (QQQ-MS/MS) or quadrupole time-of-flight high-resolution mass spectrometers (QTOF-HRMS). Non- and ^13^C-labeled key metabolites of cellular metabolism like sugar phosphates, organics acids, or amino acids can be quantified without derivatization [16] and the data analysis of the complete isotopologue space can be streamlined [17]. 

Depending on the experimental setup of the cultivation experiments, model-based approaches enable an assessment of the actual metabolic state, deriving valuable criteria, and systemic parameters. In vivo metabolic fluxes can be quantified through ^13^C metabolic flux analysis (MFA) by analyzing ^13^C-isotopologues of metabolic intermediates or endproducts [18,19], providing a basis for further strain engineering targets. In contrast, metabolic control analysis (MCA) aims to identify those enzymatic reactions that exert the highest flux control in a pathway of interest [20]. Theoretically, this is done by applying infinitesimal metabolic perturbations from steady-state conditions. However, pragmatic approaches derived metabolic control coefficients from fed-batch data using metabolic models [21,22]. Alternatively, the thermodynamically based linlog approaches [23] could be used to model metabolism kinetics and to reduce the number of model parameters [24,25]. Both of these iterative strategies require distinct modeling knowledge, which might be a challenge for some strain engineering labs and can consume high amounts of time and resources [19]. 

**Figure 1:**
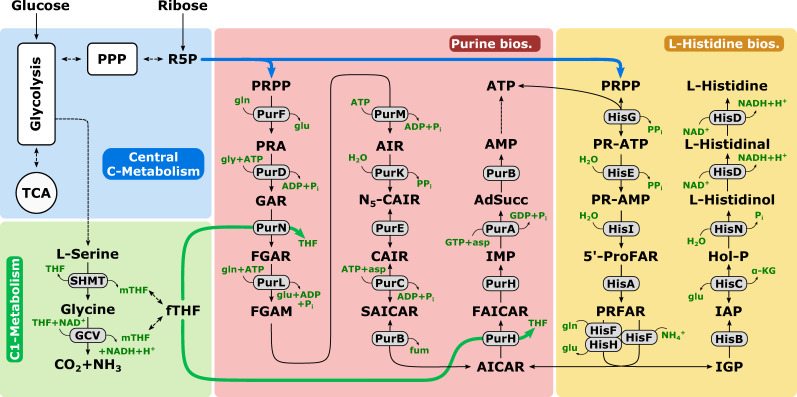
High-level overview of relevant metabolic pathways of *C. glutamicum* for the production of l-histidine. The central carbon metabolism (blue), one carbon metabolism (green), purine biosynthesis (red), and l-histidine biosynthesis (yellow) are shown. Abbreviations: 5′-ProFAR, 1-(5-phosphoribosyl)-5-[(5-phosphoribosylamino)methylideneamino] imidazole-4 carboxamide; α-KG, α-ketoglutarate; ADP, adenosine diphosphate; AdSucc, adenylosuccinate; AICAR, 1-(5′-phosphoribosyl)-5-amino-4-imidazolecarboxamide; AIR, 5-aminoimidazole ribotide; AMP, adenosine monophosphate; asp; l-aspartate; ATP, adenosine triphosphate; fTHF, 10-formyltetrahydrofolate; FAICAR, 5-formamidoimidazole-4-carboxamide ribotide; FGAM, 5′phosphoribosylformylglycineamidine; FGAR, phosphoribosyl-N-formylglycineamide; fum, fumarate; GAR, glycineamide ribonucleotide; GCV, glycine cleavage system; gln, L-glutamine; glu, l-glutamate; HisA, 5′ProFAR isomerase; HisB, imidazoleglycerol phosphate dehydratase; HisC, histidinol phosphate aminotransferase; HisD, histidinol dehydrogenase; HisE, phosphoribosyl-ATP pyrophosphatase; HisF, synthase subunit of IGP synthase; HisG, ATP phosphoribosyltransferase; HisH, glutaminase subunit of IGP synthase; HisI, phosphoribosyl-AMP cyclohydrolase; HisN, histidinol-phosphate phosphatase; Hol-P, l-histidinol phosphate; IAP, imidazole-acetole phosphate; IGP, imidazole-glycerol phosphate; IMP, inosine monophosphate; mTHF, 5,10- methylenetetrahydrofolate; N_5_-CAIR, 5′-phosphoribosyl-4-carboxy-5-aminoimidazole; NAD+/NADH, oxidized/reduced nicotine amide dinucleotide; Pi/PPi, inorganic phosphate/diphosphate; Pgm, phosphoglucomutase; PR-AMP, phosphoribosyl-AMP; PR-ATP, phosphoribosyl-ATP; PRFAR, 5-[(5-phospho-1-deoxyribulos-1-ylamino)methylideneamino]-1-(5-phosphoribosyl) imidazole-4-carboxamid; PRPP, phosphoribosyl pyrophosphate; PurA, adenylosuccinate synthase; PurB, adenylosuccinate lyase; PurC, phosphoribosylaminoimidazole- succinocarboxamide synthase; PurD, PRA-glycine ligase; PPP, pentose phosphate pathway; PurE, phosphoribosylaminoimidazole mutase; PurF, amidophosphoribosyltransferase; PurH, bifunctional AICAR formyltransferase/IMP cyclohydrolase; PurK, phosphoribosylaminoimidazole carboxylase; PurL, phosphoribosylformylglycinamide synthase; PurM, phosphoribosylformylglycinamidine cycloligase; PurN, phosphoribosylglycinamide formyltransferase; R5P, ribose 5-phosphate; SAICAR, phosphoribosyl-aminoimidazolesuccinocarboxamide; SHMT, serine hydroxymethyltransferase; TCA, tricarboxylic acid cycle; THF, tetrahydrofolate.

As an alternative to model-based techniques, purely data-driven approaches such as pool efflux capacity (PEC) may simplify and reduce modeling efforts, still producing similar results as model-based MCA approaches [26]. Even small ^13^C-based perturbations of anabolic biosynthesis pathways can be analyzed excluding the well-known drawback of metabolic leakage during cellular quenching and further streamlining the identification of strain engineering targets [27]. To showcase the application of DBTL cycles in industrial strain engineering, we generated three l-histidine producing *Corynebacterium glutamicum* strains (HIS1, HIS2, and HIS3). l-histidine is an especially challenging amino acid product due to the entwined biosynthesis with pathways such as purine biosynthesis and C_1_ metabolism (Figure 1).

**Figure 2:**
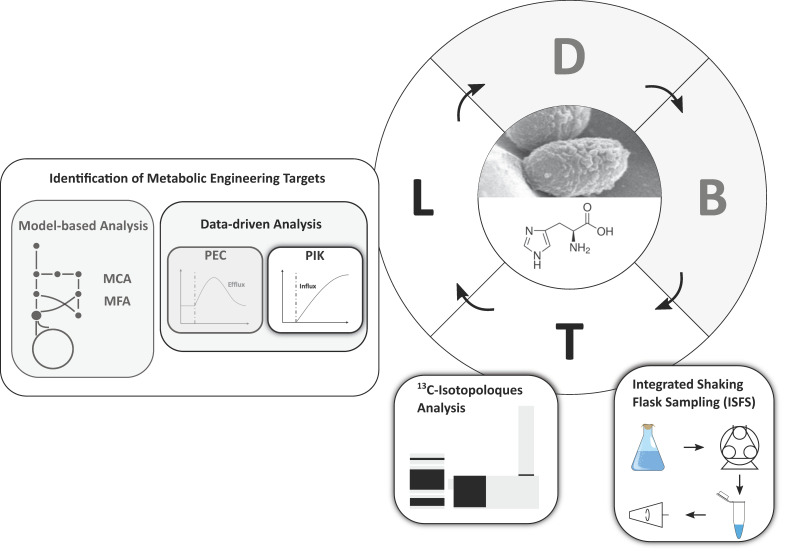
Schematic overview of the design-build-test-learn (DBTL) cycle for the l-histidine production with *C. glutamicum*. Highlighted modules were developed and applied in this work. Grey modules were not performed, but are depicted to classify the novelty of the pool influx kinetics (PIK) approach.

Both the l-histidine and purine biosynthesis share the same precursor in phosphoribosyl pyrophosphate (PRPP). Additionally, ATP is covalently incorporated in the first step of the l-histidine biosynthesis and is regenerated through 5-aminoimidazole-4-carboxamide (AICAR) in the purine biosynthesis [28]. By performing ^13^C-tracer experiments with integrated shaking flask sampling (ISFS) and subsequent HILIC-based QTOF-HRMS analytics, we show that pool influx kinetics (PIK) can be an alternative to PEC and further streamline the test and learn segments of the DBTL cycle for strain engineering (Figure 2).

## 2. Results

### 2.1. d-Ribose as a Tracer Molecule

**Figure 3:**
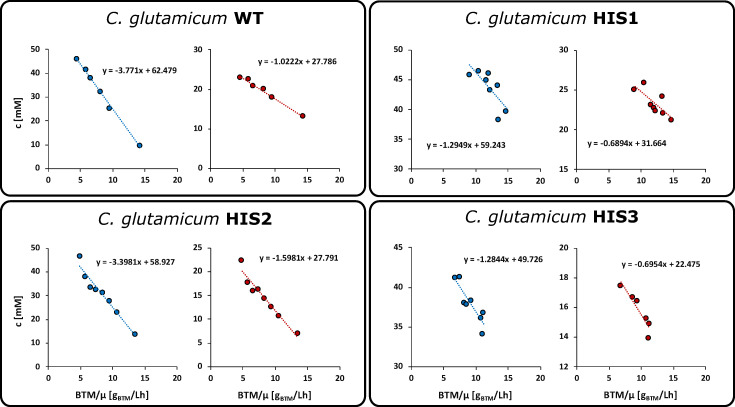
Determination of glucose (blue) and ribose (red) uptake rates. Uptake rates were calculated by linear regression on the basis of three independently performed experiments (*n* = 3).

The choice of an appropriate stimulus substrate is of high importance for the success of any tracer experiment. Ideally, the substrate is readily taken up by the production host and part of the analyzed reaction network or in close proximation to achieve sufficient perturbation. Due to the instability of relevant metabolites under cultivation conditions (e.g., PRPP [29]) or the commercial unavailability of ^13^C-labeled metabolites at the upper part of either the purine biosynthesis or the l-histidine biosynthesis, other potential tracer molecules had to be investigated. As a stimulus candidate, d-ribose was chosen due to the 2-step conversion of d-ribose to PRPP and the ability of *C. glutamicum* to simultaneously grow on d-glucose and d-ribose [30]. d-glucose and d-ribose uptake rates of the *C. glutamicum* wild-type and l-histidine producing mutants HIS1, HIS2, and HIS3 were determined by shaking flask cultivations. After an initial growth phase (4 h) with d-glucose as the sole carbon source, 20 mM d-ribose was added under exponential growth conditions. LC-QTOF-HRMS analysis of extracellular titers enabled the determination of biomass-specific uptake rates by linear regression of the concentration and biomass dry weight (BTM) divided by the growth rate (µ) [27]. Figure 3 shows the concomitant uptake of both sugars during the exponential growth of all four strains.

d-glucose was taken up with 3.8 ± 0.1 mmol g_CDW_^−1^ L^−1^ h^−1^ (WT), 1.3 ± 0.4 mmol g_CDW_^−1^ L^−1^ h^−1^ (HIS1), 3.4 ± 0.3 mmol g_CDW_^−1^ L^−1^ h^−1^ (HIS2), and 1.3 ± 0.3 mmol g_CDW_^−1^ L^−1^ h^−1^ (HIS3). d-ribose uptake rates were significantly lower with 1.0 ± 0.1 mmol g_CDW_^−1^ L^−1^ h^−1^ (WT), 0.7 ± 0.2 mmol g_CDW_^−1^ L^−1^ h^−1^ (HIS1), 1.6 ± 0.2 mmol g_CDW_^−1^ L^−1^ h^−1^ (HIS2), and 0.7 ± 0.3 mmol g_CDW_^−1^ L^−1^ h^−1^ (HIS3). Interestingly, the ratio of the d-ribose to d-glucose uptake rates changed for the mutants from 0.26 in the wild-type to 0.54 (HIS1), 0.47 (HIS2), and 0.54 (HIS3) in the producer strains. Based on these d-ribose uptake rates, the amount of ^13^C_5_-d-ribose tracer was calculated to be taken up in 20 min for subsequent labeling experiments. 

### 2.2. Integrated Shaking Flask Sampling (ISFS)

Dynamic metabolic labeling experiments are heavily dependent on fast and straight forward sampling and quenching strategies to generate representative snapshots of cellular metabolism enabling valid pool quantification and subsequent modeling and data calculations [31]. Hence, it was mandatory to develop an integrated cultivation, sampling, and quenching strategy for shaking flask cultivations to enable fast analysis of three l-histidine producing *C. glutamicum* mutants. 

**Figure 4:**
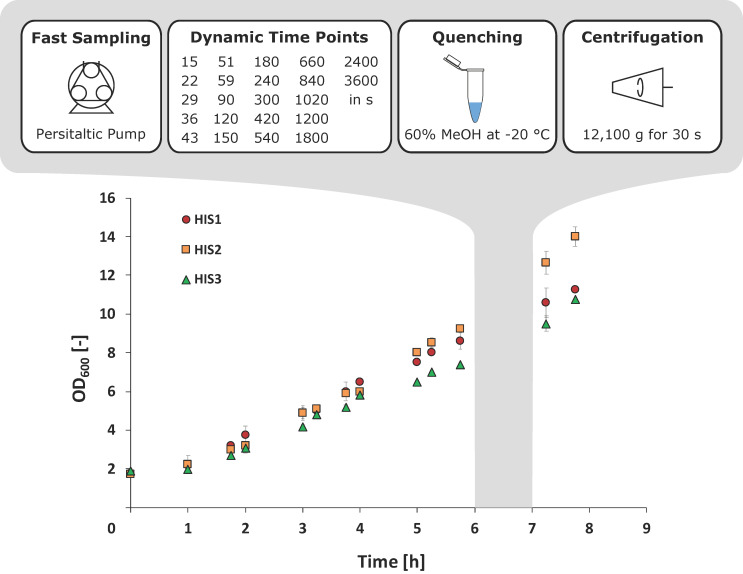
Growth curves of *C. glutamicum* HIS1, HIS2, and HIS3 during the tracer experiment and the schematic workflow of the fast sampling procedure. ^13^C_5_-ribose was added after 6 h, and sampling was finished at the seven-hour mark. Error bars give the standard deviation of three independently performed experiments (*n* = 3).

To achieve sufficiently small sampling intervals, a peristaltic pump was used, which was directly connected to a cannula inside the shaking flask. After the addition of the ^13^C_5_-d-ribose tracer, samples could be taken every seven seconds and were continuously increased up to 60 min (Figure 4). To inactivate the cellular metabolism, cell suspensions were directly transferred into 60% (v·v^−1^) methanol at −20 °C. Exponential pre-stimulus growth rates (*µ*_max_) of *C. glutamicum* HIS1 and HIS2 were 0.29 ± 0.02 h^−1^, and *µ*_max_ for HIS3 was slightly lower with 0.25 ± 0.01 h^−1^. Interestingly, the HIS2 strain clearly had the highest optical density at the end of the experiment with OD_600_ = 14.2 ± 0.5 compared to 11.3 ± 0.8 (HIS1) and 10.7 ± 0.1 (HIS3) (Figure 4).

### 2.3. HILIC-QTOF-HRMS Analytics Enables Analysis of ^13^C-Isotopologues

l-histidine biosynthesis is deeply intertwined with the central carbon metabolism, purine biosynthesis, and one-carbon metabolism (Figure 1). Due to this complexity, the analysis of the reaction network demands a different approach compared to classic linear biosynthesis pathways. 

The usage of a quadrupole time-of-flight high-resolution mass spectrometer (QTOF-HRMS) coupled to hydrophilic interaction chromatography (HILIC) enables untargeted analysis of ^13^C-labeled metabolite extracts with high spectral accuracy, sensitivity, and retrospective data mining [17]. Even computational evaluation of compounds without commercially available standards like IGP, adenylosuccinate, SAICAR, and FGAR is possible, and they can be identified by combining accurate mass and plausible fragmentation patterns [32].

**Figure 5:**
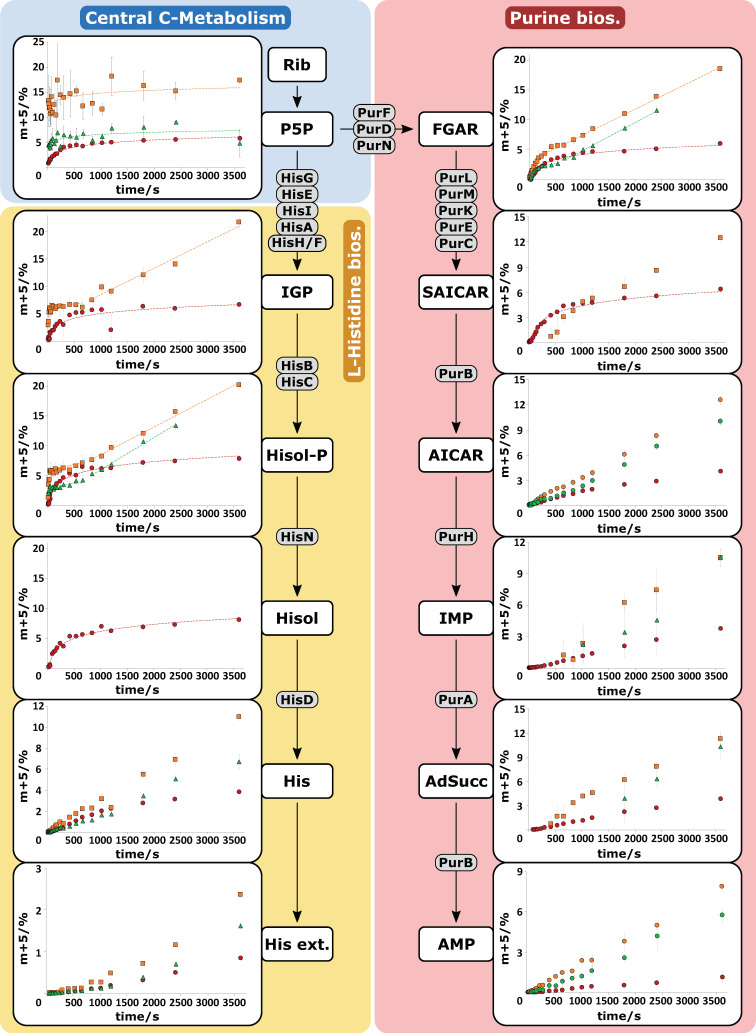
Propagation of ^13^C_5_-ribose tracer signal in *C. glutamicum* HIS1 (red dots), HIS2 (orange squares), and HIS3 (green triangles). Error bars give the standard deviation of three independently performed experiments (*n* = 3). Abbreviations: Rib, d-ribose; Hisol-P, l-histidinol phosphate; Hisol, l-histidinol; His, l-histidine; His ext., extracellular l-histidine; for other abbreviations (see Figure 1).

Using this approach, labeling patterns of twelve relevant metabolites in the l-histidine and purine biosynthesis could be automatically extracted. The ratios of m + 5 isotopologues during the sampling period of the tracer experiments for the l-histidine producing *C. glutamicum* mutants (HIS1, HIS2, HIS3) are depicted in Figure 5. Since not all metabolites in the two linear pathways of the biosynthesis network were commercially available as a reference standard, several reaction steps were fused to a total conversion, whereby the involved enzymes are shown in grey in Figure 5. 

The initial endogenous metabolite ribose 5-phosphate is labeled by phosphorylation of d-ribose inside of the cell. Since ribose 5-phosphate, ribulose 5-phosphate, and xylulose 5-phosphate cannot be distinguished by retention time and accurate mass (isobaric analytes), these metabolites are shown as the pentose 5-phosphate pool (P5P) [16]. The lumped pool was rapidly labeled and converged toward 5% m + 5 isotopologue for HIS1 and HIS3 and 15% m + 5 for HIS2, which reflects the different d-ribose uptake rates for the three strains. The sampling intervals were sufficient to depict the initial labeling of the P5P pool for HIS1, but not for HIS2 and HIS3. However, the reaction cascade showed clear propagation of the ^13^C-labeling signal (Figure 5, left side), and the stimulus-induced labeling dynamics were widely resolved. Some metabolites like Hisol and IGP were not detected in every mutant strain due to pool levels below the detection limit. Strikingly, tracer propagations leveled out for the metabolites IGP, Hisol-P, and Hisol in all three *C. glutamicum* mutants, whereby HIS2 and HIS3 had postponed linear labeling dynamics.

As a recycling pathway for AICAR, which is produced in the l-histidine biosynthesis, the purine biosynthesis plays an essential role in the biotechnological production of l-histidine with *C. glutamicum* [32]. As PRPP is the common precursor for both pathways (Figure 1), simultaneous labeling of the pathways was achieved. Analyzed intermediates of the purine pathway (Figure 5, right side) showed comparable labeling dynamics. Related to some targets in the l-histidine biosynthesis, SAICAR also did not reveal labeling in all three mutants due to pool levels below the detection limit. As seen before, multiple subsequent dynamics were observed in the metabolites of the purine biosynthesis like FGAR and SAICAR.

Taken together, the ISFS approach, combined with HILIC based QTOF-HRMS analytics, enabled the analysis of the complete isotopologue space of twelve metabolites (*n* = 3) in a fast fashion using automated data processing. 

### 2.4. Pool Influx Kinetics as a Metabolic Engineering Tool

**Figure 6:**
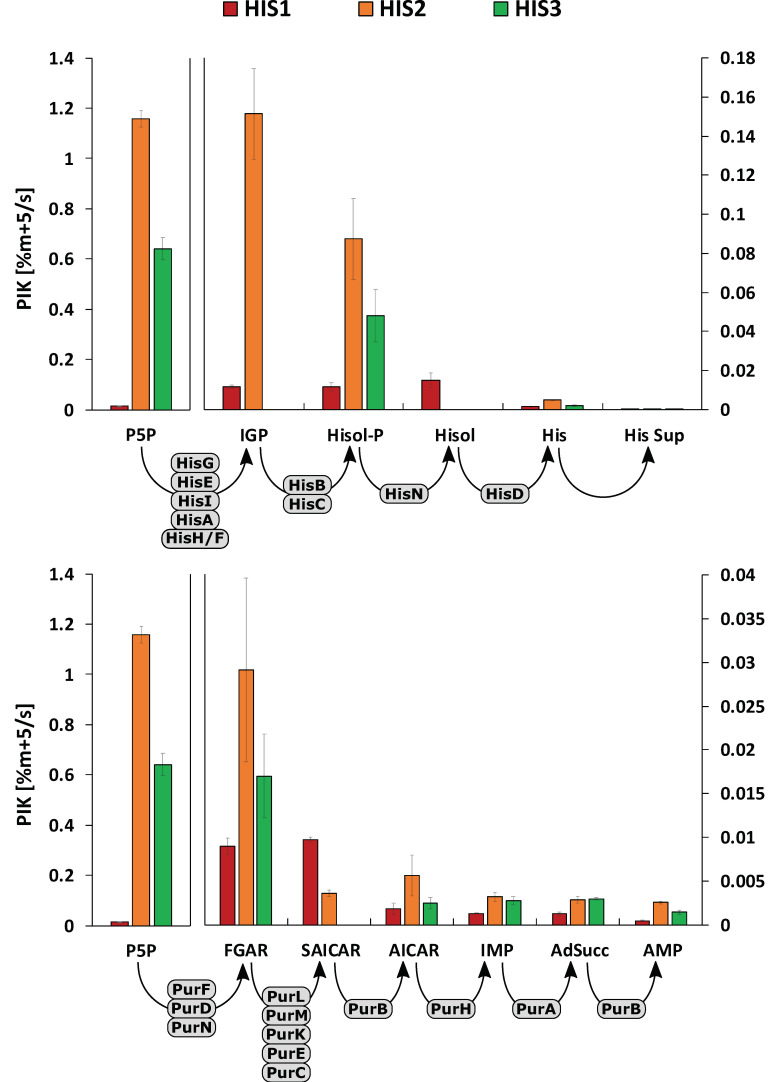
Pool influx kinetics (PIK) of measured metabolites in the l-histidine biosynthesis (**upper**) and purine biosynthesis (**lower**). Standard deviations are given as error bars. Corresponding enzymes are depicted in grey. PIK values were based on the experiments of three biological replicates (*n* = 3).

To demonstrate a new technique for the Learn segment of the DBTL cycle, PIK values were calculated, and the results were visualized as bar diagrams for both linear reaction pathways (Figure 6). Of note, the PIK values indicate the percentage of labeling enrichment (here: m + 5) per second. Consequently, the values can be qualified as a proxy for the labeling velocity (i.e., the upstream supply of labeling information via reaction rates) by assuming that metabolite pools are at pseudo-stationary conditions during the observation window. Accordingly, high PIK values mirror fast upstream reaction, whereas low PIKs identify low reaction rates. The latter may enable the identification of metabolic engineering targets if up- and downstream PIK values differ significantly from each other. 

As a common precursor of the pathways, P5P is part of both diagrams, and the value has separated scaling of the y-axis because of the extremely high PIKs compared to the succeeding metabolites. For the mutant strains, HIS2 and HIS3 PIK values were calculated differentially due to the rapid labeling of the P5P pool. As the first step, HIS1 was analyzed to identify further metabolic engineering targets. The strain already possessed several modifications in the l-histidine biosynthesis pathway (Table 1, [32]). Early response (<500 s) PIK values for the purine biosynthesis (Figure 6) showed a 5-fold decrease from SAICAR to AICAR, pinpointing to delayed signal transduction. 

Looking at the subsequent metabolites in the linear pathways, this trend continued with a 7-fold (IMP), 7-fold (AdSucc), and 20-fold (AMP) decrease in PIK values compared to SAICAR. The corresponding enzymes for these reactions are PurA, PurB, and PurH (Figure 1), which consequently could be identified as strain engineering targets. Based on these findings, the DBTL cycle was completed. A new optimization round started engineering HIS2 strain [32], which incorporates plasmid-encoded amplification of the native *purA* and *purB* genes. Strikingly, this strain does not achieve a higher l-histidine yield than HIS1. Nevertheless, another PIK analysis was performed to qualify the current flux control. As intended by *purA* and *purB* amplification, PIK values of AICAR, IMP, AdSucc, and AMP all increased by 3-fold, 2.4-fold, 2.2-fold, and 5.5-fold, respectively, compared to HIS1. As previously described, ATP is covalently bound to PRPP in the first reaction of the l-histidine biosynthesis. Subsequently, the nucleotide backbone is recycled as AICAR in the purine biosynthesis by the reaction sequence of AICAR → FAICAR → IMP → AdSucc → AMP. Interestingly, there was a 40% drop in PIK values between AICAR and the subsequent metabolites, identifying the reaction sequence from ACIAR → IMP as another strain engineering target. In this sequence, AICAR is converted to FAICAR by the enzyme PurH with the use of the cofactor 10-formyltetrahydrofolate fTHF. Next, FAICAR reacts to IMP catalyzed by PurH (Figure 1). Based on the PIK values, there are two potential ways to improve this reaction sequence, either at the enzyme level or supplying more cofactor. It was decided to test the improved supply of the cofactor fTHF, which was considered as the next DBTL cycle.

Based on the HIS2 strain, HIS3 was engineered harboring a second plasmid with the GCV system from *Corynebacterium jeikeium*. Through the introduction of GCV, the supply of 5,10-methylenetetrahydrofolate (mTHF), and finally, fTHF was improved by enhanced recycling of the glycine pool. As a consequence, a higher l-histidine yield compared to *C. glutamicum* HIS2, and no secretion of glycine into the medium was observed [32]. Interestingly, this strain showed similar IMP PIK values (0.0032 ± 0.0005% (m + 5) s^−1^) than HIS2 (0.0028 ± 0.0005% (m + 5) s^−1^), even though the availability of fTHF should have been increased. Noteworthy, fTHF is also needed by PurN, producing the purine biosynthesis intermediate FGAR (Figure 1). Concomitantly, no SAICAR labeling patterns were measured in *C. glutamicum* HIS3 because they were below the detection limits. Concluding this DBTL cycle, the amplification of PurH remains as a potential strain engineering target. 

However, future strain engineering efforts could also focus on the linear reaction network of the l-histidine biosynthesis. Figure 6 depicts 7-fold (HIS1), 181-fold (HIS2), and 22-fold (HIS3) PIK value decreases from Hisol-P to His and 61-fold (HIS1), 1711-fold (HIS2), and 279-fold (HIS3) decrease from Hisol-P to His Sup. Apparently, improving l-histidine export would be a proper metabolic engineering target, although no distinct l-histidine exporter is known so far in any organism. Still, homo- or heterologous expression of HisN or HisD might be promising targets to increase PIK values in l-histidine biosynthesis. Summarizing, the second DBTL cycle pinpointed to multiple metabolic engineering targets dealing with (i) PurH amplification, (ii) debottlenecking l-histidine biosynthesis, and (iii) improving l-histidine export. These should be topics of subsequent DBTL cycles.

## 3. Discussion

Systematic testing of engineered producer strains for qualifying genetic manipulations and for deriving novel hypothesis for the next DBTL cycle is an equally crucial and demanding task that is often laborious and time-consuming. A thorough metabolic analysis may require multiple weeks [19], whereas recent developments speed up molecular cloning and DNA design to almost a single day [33]. 

In this work, we presented a straightforward data-driven approach, defining pool influx kinetics (PIK) for identifying reactions that exert high flux control in linear pathways. PIK analyses follow the idea of pool efflux capacities (PEC) by ranking the net reaction rates of metabolite pools after non-stationary ^13^C-tracer perturbation [26,27]. Highly differing pool influx and efflux rates refer to promising enzymatic targets for rational strain design. The excellent performance of HILIC-QTOF-HRMS analytics [17] measuring dynamic ^13^C-labeling patterns (perturbations) with subsequent automated data extraction streamlines the test and learn segments of the DBTL cycles significantly. 

However, the reduction of perturbation strengths in complex and strictly regulated metabolic networks (signal dilution) demands sophisticated stimulus strategies [27,34]. A priori studies identify ^13^C_5_-d-ribose as an informative and cost-efficient full-labeled stimulus substrate closely related to PRPP as a common precursor for l-histidine and purine biosynthesis. The rapid and concomitant uptake of d-ribose and d-glucose by a ribose-specific ABC transporter showed no catabolite repression [30], but enhanced consumption rates in histidine producers, which correlates with the higher carbon demands for fueling downstream fluxes toward l-histidine. Interestingly, the d-ribose to d-glucose uptake ratios stayed virtually the same with around 0.5 for all producer strains, pointing toward similar central carbon flux distributions, irrespective of engineered targets in the biosynthesis routes. However, the latter impaired maximal growth rates in l-histidine producers [32], apparently because precursors for biomass formation were drained. 

Instationary ^13^C-based metabolic analysis has the advantage that the time of the actual experiment can be reduced significantly compared to stationary analysis [19,35,36,37]. However, high sampling frequencies, especially at the beginning of the experiments, become more critical to monitor fast labeling dynamics, often occurring in central carbon metabolism [38]. By developing the integrated shaking flask sampling approach (ISFS) with immediate cold methanol quenching by a peristaltic pump, minimal intervals of seven seconds were achieved with a limited and cost-efficient automation effort. Since further analysis focused on ratios of different ^13^C-isotopologues instead of absolute concentrations of metabolites, the drastic unspecific leakage of metabolites, which was previously described for *C. glutamicum* [39], did not affect the outcome of this study. However, even faster sampling would have been necessary to resolve the dynamics of the P5P pool in strain HIS2 and HIS3 and demands further optimization. Next, the workload for the cultivation experiments was minimized by using shaking flasks instead of technically demanding bioreactor fermentations, which enabled the time-efficient analysis of three different l-histidine production hosts in biological triplicates. Accordingly, the approach should be readily applicable in multiple labs without requiring sophisticated technical bioreactor equipment and dedicated skills to run fermentation tests properly.

Time and manual operations were also saved by analyzing the metabolite extracts with HILIC-QTOF-HRMS. Compared to other approaches, no sample derivatization [40] or analytical fragmentation [41] of metabolites was necessary. This setup allowed the analysis of the isotopologue space of twelve metabolites in one single run and could be extended to up to more than 60 hydrophilic metabolites of cellular metabolism in future works [16]. Thanks to the commercially available software, isotopologues were automatically extracted with natural isotopic background subtraction. The subsequent analysis focused on the m + 5 isotopologue of corresponding metabolites reaching sufficient labeling ratios (3–20%) within the perturbation period. However, various other isotopologues like m + 3, m + 2, or m + 1 were observed for some metabolites due to reshuffling in the non-oxidative part of the pentose phosphate pathway (data not shown). Notably, analyzing metabolite signals multiple steps downstream of signal induction usually comes along with severe signal dilution, which could be generally problematic for analyzing anabolic reaction networks with low sensitivity analytics [34].

The ^13^C_5_ tracer amount was a priori designed for a perturbation period of 20 min, reflecting allosteric regulation of the reaction network at a metabolic level [26]. As shown in Figure 5, actual stimulus periods were significantly extended (up to 60 min), which is presumably based on transient and concentration-dependent d-ribose uptake rates. Consequently, any kind of labeling analysis requiring a constant uptake rate during a long-term window of labeling is likely to fail. In contrast, the idea of pool influx kinetics (PIK) only requires the comparison of up- and downstream labeling dynamics, even analyzing short time windows. Here, initial labeling dynamics of the first 500 s well fulfilled the constraint and provided the basis for DBTL cycling due to the short time scale. However, some metabolites showed labeling dynamics at the end of the labeling period, which could be due to an interfering regulation on the transcriptional level [42].

Identifying proper metabolic engineering targets is a key issue of the learn segment in the DBTL cycle. Here, we proposed the iterative use of PIK values to identify targets for the next round of cell design. As described before [32], the starting strain HIS1 revealed an impaired energy metabolism with strongly diminished adenosine nucleotide (AxP) levels, mainly based on an accumulation of IMP. The phenomenon is well reflected in the comparison of PIK values for the P5P pool in HIS1 (0.013 ± 0.002% (m + 5) s^−1^) and also in HIS2 (1.158 ± 0.033% (m + 5) s^−1^). Apparently, both strains have very low activity of the pentose phosphate pathway. 

The DBTL cycle started with HIS1, identifying the targets PurA, PurB, and PurH by qualifying the drastic PIK reduction from SAICAR to AICAR as a limited precursor supply due to the inability of the cell to recycle ATP. By introducing an overexpression plasmid of the native *purA* and *purB* genes into *C. glutamicum* HIS1, AxP levels improved, and PIK values of the metabolites downstream of SAICAR also significantly rose. Even though the metabolic engineering strategy successfully improved the precursor supply of AICAR, HIS2 did not achieve increased l-histidine yields (0.054 ± 0.002 mol l-histidine per mol d-glucose). These results shifted the focus to so far not addressed reaction networks as C_1_ metabolism. In particular, the availability of the cofactor fTHF was indirectly enhanced by inserting a plasmid harboring the GCV system into HIS2, which represented the second optimization round. The yield could be increased to 0.086 ± 0.001 mol l-histidine per mol d-glucose, reflecting the diminished by-product formation of glycine as previously observed for other l-histidine producing mutants of *C. glutamicum* and *Brevibacterium flavum* [43,44].

Interestingly, PIK values of FGAR and IMP, both downstream of formyl integration from fTHF, did not improve as expected. Consequently, one may conclude that fTHF supply still deserves further amplification to boost the carbon flux into l-histidine. However, in-depth quantitative analysis of the various THF species of the C_1_ metabolism is not possible due to low pool sizes caused by interconversion, polyglutamylation, and degradation [45]. 

Nevertheless, the amplification of *purH*, which was already on the task list of the first round of DBTL cycling, still remains as a highly promising metabolic engineering target. The importance of PurH debottlenecking as even increased by analyzing the PIK values of AICAR: Given that labeling signals of ribose are exclusively transferred into IGP and that ATP only revealed very poor labeling intensity (data not shown), AICAR produced by the l-histidine biosynthesis was also poorly labeled. In contrast, AICAR originating from purine biosynthesis should receive fast labeling transfers from SAICAR. Strikingly, downstream reactions of AICAR leading to IMP only disclosed very low PIK values of 0.0013 ± 0.0003% (m + 5) s^−1^ (HISF1) and 0.0032 ± 0.004% (m + 5) s^−1^ (HISF2), which correspond to decreasing PIK values of 9-fold (HISF1) and 47-fold (HIS2) with respect to AICAR. The substantial slow-down of labeling signal transduction may be interpreted by (i) low downstream rates catalyzed by PurH and (ii) dominating carbon fueling from the non-labeled AICAR originating from l-histidine biosynthesis. The latter requires further engineering work to equilibrate carbon fluxes in the purine and l-histidine biosynthesis. The necessity of amplifying fluxes through PurH has already been outlined. Additional consideration deserves the finding that accumulation of AICAR was identified to inhibit HisG [46] competitively and bind to a riboswitch, which negatively controls the expression of purine genes [47] in *E. coli*. Consequently, PurH engineering remains the prime task for further strain optimization.

In summary, the PIK concept is not exclusive for the analysis of the l-histidine pathway. The integrated approach, comprising cultivation, sampling, and HILIC-QTOF-HRMS based instationary ^13^C tracer analysis, offers fast DBTL cycling to identify promising metabolic engineering targets in linear pathway segments. However, further correlation analysis with classical ^13^C MFA approaches needs to be done to determine the suitability of PIK as a metric for flux indication. Additional improvements in analytical equipment and the expansion of the analyzed metabolites to a more holistic system-wide approach paired with even faster sampling and simplified cultivation will elevate testing and learning for future industrial strain engineering.

## 4. Materials and Methods 

### 4.1. Media and Cultivation Conditions

All relevant strains for this study were introduced and published previously [32] and are shown in Table 1. Shaking flask cultivations of *C. glutamicum* WT, HIS1, HIS2, and HIS3 were carried out in biological triplicates (*n* = 3, 50 mL), as described in [32] with the exception that the optical density at 600 nm (OD_600_) at the start of the cultivation of the main culture was 2.0. The maximal growth rate *µ*_max_ was calculated by the linear regression of ln(OD600) plotted against the cultivation time in h during the exponential growth phase of the respective strain.

### 4.2. Chemicals

Chemical reagents (>99% p.a) and ^13^C_5_-d-ribose (99 atom%) were bought from Sigma–Aldrich (Taufkirchen, Germany). MS-grade water was purchased from VWR (Darmstadt, Germany). MS-grade acetonitrile and HPLC-grade methanol were purchased from Carl Roth (Essen, Germany). Standard stock solutions were prepared in LC-MS water and stored at −70 °C. 

### 4.3. Fast Sampling Procedure and Methanol Quenching

^13^C tracer experiments were carried out with fully labeled ^13^C_5_-d-ribose as a stimulus substrate. After six hours of shaking flask cultivation, the ^13^C_5_-d-ribose stimulus was added via a sterile syringe. Sampling was immediately performed, while shaking, by using a peristaltic pump (120U, Watson-Marlow, Falmouth, UK) and in adapted time intervals. For metabolic inactivation, 1 mL of suspension culture was directly quenched in 1.5 mL 60% (v∙v^−1^) methanol precooled at −60 °C in 5 mL Eppendorf tubes and centrifuged for 30 s at 4000× *g* and −11 °C (5430R, Eppendorf, Hamburg, Germany). Additionally, 0.5 mL of analogous suspension culture was directly centrifuged for 20s at 20,000× *g* and room temperature (5417R, Eppendorf, Hamburg, Germany) for supernatant analysis. Quenched biomasses and supernatants were shock frozen by liquid nitrogen (−196 °C) and temporarily stored at −70 °C. 

### 4.4. Metabolite Extraction

For the extraction of metabolites, defined amounts (80–120 μL) of 300 μM l-norvaline solution (internal standard) were added to the stored cell pellets to obtain an extraction concentration of 20 g_CDW_∙L^−1^ (CDW = OD_600_ × 0.21 g∙L^−1^). The resulting cell suspension was immediately incubated for 30 s at 100 °C in a water bath, vortexed for 20 s, and again incubated for 3 min at 100 °C. Subsequently, the lysed cell suspension was centrifuged for 10 min at 20,000× *g* and 4 °C (5417R, Eppendorf, Hamburg, Germany). The separated metabolite extracts were stored at −70 °C until further analysis.

### 4.5. LC-QTOF Analysis

#### 4.5.1. Analysis of d-Glucose and d-Ribose in Supernatant Samples

Quantification of monosaccharides was performed on an Agilent 1260 bio-inert HPLC coupled to an Agilent 6540 Accurate-Mass LC-MS/MS Q-TOF system with ESI Jet Stream Technology (Agilent Technologies, Santa Clara, CA, USA). The developed method utilized a Waters XBridge BEH Amide column (150 × 2.1 mm, 3.5 μm) coupled to a Waters XBridge BEH Amide VanGuard Cartridge (Waters, Milford, OH, USA) (5 × 2.1 mm, 3.5 μm) at 35 °C, 0.2 mL min ^−1^, and 5 μL injection volume. Samples were diluted 1:2000 with water and prepared in 60% (v·v^−1^) acetonitrile and 10 mM ammonium acetate (pH 9.2). Mobile phase compositions of the quaternary pump system were as follows, A: 100% acetonitrile, B: 10% acetonitrile/90% water, C: 100% methanol, D: 400 mM ammonium acetate pH 9.2 (adjusted with 25% (v v^−1^) ammonium hydroxide). Gradient elution was carried out according to Table 2:

All samples were analyzed in negative ESI mode (tuned in extended dynamic range MS mode), and the parameters were as follows: drying gas flow rate of 8 L min^−1^ with a gas temperature of 260 °C, nebulizer with 40 lb per square inch gauge, sheath gas flow rate of 12 L min^−1^ with a gas temperature of 340 °C, capillary voltage of 4000 V, and fragmentor voltage of 100 V. During analysis, online mass calibration was ensured by continuous introduction of reference masses (*m/z* 119.03632 and *m/z* 980.016375). System control and acquisition were performed using MassHunter Data Acquisition (B.06.01, Agilent Technologies, Santa Clara, CA, USA). Peak extraction and integration were done in MassHunter Qualitative Analysis (B.07.00, Agilent Technologies, Santa Clara, CA, USA). 

#### 4.5.2. Analysis of ^13^C-Labeled Intracellular Metabolite Extracts and Data Mining

The LC-QTOF analysis of l-histidinol phosphate (Hisol-P), l-histidinol (Hisol) l-histidine (His), imidazole-glycerol phosphate (IGP), adenylosuccinate (AdSucc), 1-(5′-phosphoribosyl)-5-amino-4-imidazolecarboxamide (AICAR), phosphoribosyl-N-formylglycineamide (FGAR), inosine monophosphate (IMP), phosphoribosyl-aminoimidazolesuccinocarboxamide (SAICAR), adenosine monophosphate (AMP), and the pentose 5-phosphate pool (R5P) was carried out as previously described [17]. Automated data analysis with the “Batch Isotopologue Extraction” feature and natural isotopologue background subtraction was carried out in the MassHunter ProFinder (B.08.00, Agilent Technologies, Santa Clara, CA, USA) software. The relative isotopologue abundances (RIA) of the m + 5 isotopologue for the twelve measured metabolites for every time point was calculated according to:

(1) $$RIA_{m+5}\left[\%\right]=\frac{Intensity\;m+5}{\sum_{j=0}^{n}Intensity\;m+j}\;n=amount\;of\;carbon\;atoms$$

Throughout the analysis, the internal standard l-norvaline was monitored to investigate the possible intensity drifts on a global MS level.

### 4.6. Pool Influx Kinetics

Pool influx kinetics (PIK) is a straightforward, purely data-driven approach for the analysis of linear pathway segments and is based on the concept of pool efflux capacities (PEC) [26,27]. In contrast to the ranking maximum net efflux rates of perturbed metabolite pools, this approach focuses on the immediate and instationary response to the influx (stimulus) after the application of a ^13^C-labeled tracer.

(2) $$PIK\left[\%\;s^{-1}\right]=\frac{d\left(m+n\right)/m_{total}\;\left[\%\right]}{dt\;\left[s\right]}$$

Equation (2) shows that the PIK value can be calculated as linear regression of the isotopologue percentage of choice (*m* + *n*)/*m*_total_ over time t. In this work, this was done based on at least four data points at the beginning of the first linear increase (R^2^ > 0.9) of the m + 5 isotopologue of each metabolite.

## Figures and Tables

**Table 1:** Strains that were used in this work and corresponding l-histidine product substrate yields (Y_P/S_^his^).

Strains	Description	Y_P/S_^his^ [mol mol^−1^]	Comment
*C. glutamicum* WT	Wildtype strain ATCC 13032	-	-
*C. glutamicum* HIS1	*C. glutamicum hisG*^G233H-T235Q^ P*_tuf_* (*hisD-hisC-hisB*-cg2302-cg2301) P*_tuf_* (*hisH-hisA-impA*-P*_sodA_*(*hisF-hisI*-cg2294)) P*_tuf_*(cg0911-*hisN*) P*_dapA_*_-A16_ (*hisE*^ATG^-*hisG*^G233H-T235Q^)	0.065 ± 0.004	HIS7 in [32]
*C. glutamicum* HIS2	*C. glutamicum* HIS1 containing pJC4 *purA purB*	0.054 ± 0.002	HIS8 in [32]
*C. glutamicum* HIS3	*C. glutamicum* HIS2 containing pEC-XT99A_gcv_OP1-Cjk	0.086 ± 0.001	HIS9 in [32]

**Table 2:** Gradient timetable of the LC-QTOF method for monosaccharide analysis.

Time (min)	A (%)	B (%)	C (%)	D (%)
0	89.75	2.75	5	2.5
36	62.25	30.25	5	2.5
40	1.75	90.75	5	2.5
45	1.75	90.75	5	2.5
50	89.75	2.75	5	2.5
70	89.75	2.75	5	2.5
